# Dropsy of the Amnion

**Published:** 1852-06

**Authors:** W. H. Pitts

**Affiliations:** Albemarle


*Dropsy of the Amnion.* By W. H. Pitts, M. D., of Albemarle.—During the afternoon of 3d February, I received an urgent call to visit a negro girl of Mr. B. H. B., aged about 17, some 6 miles distant from my location. When I arrived, and before I entered the quarter in which the girl was, her mistress informed me that she was taken on the Friday morning previous, with what they supposed were labor pains; that they sent for a midwife in the neighborhood, who pronounced their conjeetures correct; the girl however protested that it could not be so, for it was not her time as yet—that she didn't look until the middle of March. Her pains, weak and irregular, continued during that day and throughout Saturday, Sunday, Monday, and Tuesday, sometimes attributing her symptoms to colic, then to hysteria, until at length their attention was called to the very perceptible and gradually increasing enlargement of the abdomen, and the constantly increasing general distress consequent thereon—then it was that they at once determined to seek further aid.

When I entered the house, I was struck with the enormous enlargement of the abdomen; placing my hand upon it, it was found to be very tense and tender to pressure. An examination per vaginam was instituted, which revealed an os tincæ dilated to about the size of a quarter dollar, through which a tense bag of waters could be felt. Ballottement at once rendered the diagnosis clear without a chance of doubt. Her pulse frequent and feeble, being 120—respiration short and hurried—skin cool—general restlessness of system—determined me to proceed without delay to the relief of the patient. Upon inquiry, it was ascertained that the girl had passed no urine for some 18 hours, though she had recently made the attempt several times without avail. This induced me to make an effort to evacuate that viscus, but owing to the great pressure upon it by the distended uterus, it was found impossible to introduce the catheter farther than 2½ inches. I then proceeded to evacuate the uterus of its contents by rupturing the bag of waters with the point of my finger nail. It was now 5½ o'clock, P. M.; at 6 the bag, during a contraction, burst, and from what escaped into the vessel and on the floor, and afterwards during the process of the labor, I hesitate not to say, that at least 7 or 8 quarts of water escaped. The abdominal distension was at once relieved—her breathing became natural—pulse fuller and at the rate of 112—labor pains active and regular—and at 11½, P. M., a living fœtus was expelled; it seemed to be of 7 months development, was very puny and attenuated—it lived three days. I examined the appendages very minutely, but perceived nothing abnormal about them.

The patient, after the labor, expressed herself as quite comfortable—the pulse at 3 A. M., had fallen to 88, full and soft—I saw her again on the next day at 4 P. M. She expressed herself as free from pain—had slept throughout the day—the pulse had now assumed its natural character—and from this time henceforward she convalesced rapidly.

The etiology and pathology of amniotic dropsy I leave to the learned in the profession, who can enlighten us.

*North Garden, Feb.* 20, 1852.

*Stethoscope.*

